# Fostering Multidisciplinary Collaboration in Artificial Intelligence and Machine Learning Education: Tutorial Based on the AI-READI Bootcamp

**DOI:** 10.2196/83154

**Published:** 2025-12-29

**Authors:** Taiki W Nishihara, Fritz Gerald P Kalaw, Adelle Engmann, Aya Motoyoshi, Paapa Mensah-Kane, Deepa Gupta, Victoria Patronilo, Linda M Zangwill, Shahin Hallaj, Amirhossein Panahi, Garrison W Cottrell, Bradley Voytek, Virginia R de Sa, Sally L Baxter

**Affiliations:** 1 Viterbi Family Department of Ophthalmology and Shiley Eye Institute, Hamilton Glaucoma Center, Division of Ophthalmology Informatics and Data Science, University of California, San Diego, 9415 Campus Point Drive, La Jolla, CA, 92093, United States, 1 858-534-8413; 2 Department of Medicine, Division of Biomedical Informatics, University of California, San Diego, La Jolla, CA, United States; 3 Stanford Deep Data Research Center, Department of Genetics, Stanford University, Palo Alto, CA, United States; 4 Department of Ophthalmology, University of Washington, Seattle, WA, United States; 5 School of Pharmacy, South University, Savannah, GA, United States; 6 Department of Cognitive Science, University of California, San Diego, La Jolla, CA, United States; 7 Halıcıoğlu Data Science Institute, University of California, San Diego, La Jolla, CA, United States; 8 Department of Computer Science and Engineering, University of California, San Diego, La Jolla, CA, United States; 9 Neurosciences Graduate Program, University of California, San Diego, La Jolla, CA, United States; 10 Kavli Institute for Brain and Mind, University of California, San Diego, La Jolla, CA, United States

**Keywords:** artificial intelligence, machine learning, biomedical research, interdisciplinary training, data science, curriculum development, translational research, medical education

## Abstract

**Background:**

The integration of artificial intelligence (AI) and machine learning (ML) into biomedical research requires a workforce fluent in both computational methods and clinical applications. Structured, interdisciplinary training opportunities remain limited, creating a gap between data scientists and clinicians. The National Institutes of Health’s Bridge to Artificial Intelligence (Bridge2AI) initiative launched the Artificial Intelligence–Ready and Exploratory Atlas for Diabetes Insights (AI-READI) data generation project to address this gap. AI-READI is creating a multimodal, FAIR (findable, accessible, interoperable, and reusable) dataset—including ophthalmic imaging, physiologic measurements, wearable sensor data, and survey responses—from approximately 4000 participants with or at risk for type 2 diabetes. In parallel, AI-READI established a year-long mentored research program that begins with a 2-week immersive summer bootcamp to provide foundational AI/ML skills grounded in domain-relevant biomedical data.

**Objective:**

To describe the design, iterative refinement, and outcomes of the AI-READI Bootcamp, and to share lessons for creating future multidisciplinary AI/ML training programs in biomedical research.

**Methods:**

Held annually at the University of California San Diego, the bootcamp combines 80 hours of lectures, coding sessions, and small-group mentorship. Year 1 introduced Python programming, classical ML techniques (eg, logistic regression, convolutional neural networks), and data science methods, such as principal component analysis and clustering, using public datasets. In Year 2, the curriculum was refined based on structured participant feedback—reducing cohort size to increase individualized mentorship, integrating the AI-READI dataset (including retinal images and structured clinical variables), and adding modules on large language models and FAIR data principles. Participant characteristics and satisfaction were assessed through standardized pre- and postbootcamp surveys, and qualitative feedback was analyzed thematically by independent coders.

**Results:**

Seventeen participants attended Year 1 and 7 attended Year 2, with an instructor-to-student ratio of approximately 1:2 in the latter. Across both years, postbootcamp evaluations indicated high satisfaction, with Year 2 participants reporting improved experiences due to smaller cohorts, earlier integration of the AI-READI dataset, and greater emphasis on applied learning. In Year 2, mean scores for instructor effectiveness, staff support, and overall enjoyment were perfect (5.00/5.00). Qualitative feedback emphasized the value of working with domain-relevant, multimodal datasets; the benefits of peer collaboration; and the applicability of skills to structured research projects during the subsequent internship year.

**Conclusions:**

The AI-READI Bootcamp illustrates how feedback-driven, multidisciplinary training embedded within a longitudinal mentored research program can bridge technical and clinical expertise in biomedical AI. Core elements—diverse trainee cohorts, applied learning with biomedical datasets, and sustained mentorship—offer a replicable model for preparing health professionals for the evolving AI/ML landscape. Future iterations will incorporate additional prebootcamp onboarding modules, objective skill assessments, and long-term tracking of research engagement and productivity.

## Introduction

Artificial intelligence (AI) has demonstrated transformative potential in health care, with deep learning algorithms now able to screen for diabetic retinopathy from fundus photographs and predict patient-specific glycemic fluctuations [1] at performance levels comparable to expert clinicians. However, despite these advances, a persistent gap remains between model developers and clinical end users.

Clinicians often lack formal training in AI and machine learning (ML) or data science. Only about 28% of published AI/ML model development studies include clinician involvement, and their contributions are frequently limited [2]. Likewise, in the United Kingdom, just 13.8% of trainee physicians reported feeling adequately prepared for the integration of AI into clinical practice [3]. Conversely, engineers and computer scientists are rarely trained in the clinical, regulatory, or ethical complexities of health care delivery. This persistent disconnect constrains interdisciplinary collaboration, limits translational impact, and risks generating AI systems that perform poorly in real-world clinical settings [4-6].

Recognizing these interdisciplinary and workforce gaps, the National Institutes of Health (NIH) launched the Bridge to Artificial Intelligence (Bridge2AI) initiative in 2022 to promote the creation of FAIR (findable, accessible, interoperable, and reusable) multimodal datasets while advancing coordinated skills and workforce development [7]. Among its flagship data generation projects (DGPs), the Artificial Intelligence–Ready and Exploratory Atlas for Diabetes Insights (AI-READI) is curating a comprehensive dataset integrating ophthalmic imaging, physiologic measurements, wearable sensor data, and survey responses from approximately 4000 individuals with or at risk for type 2 diabetes [8,9].

**Figure 1.**
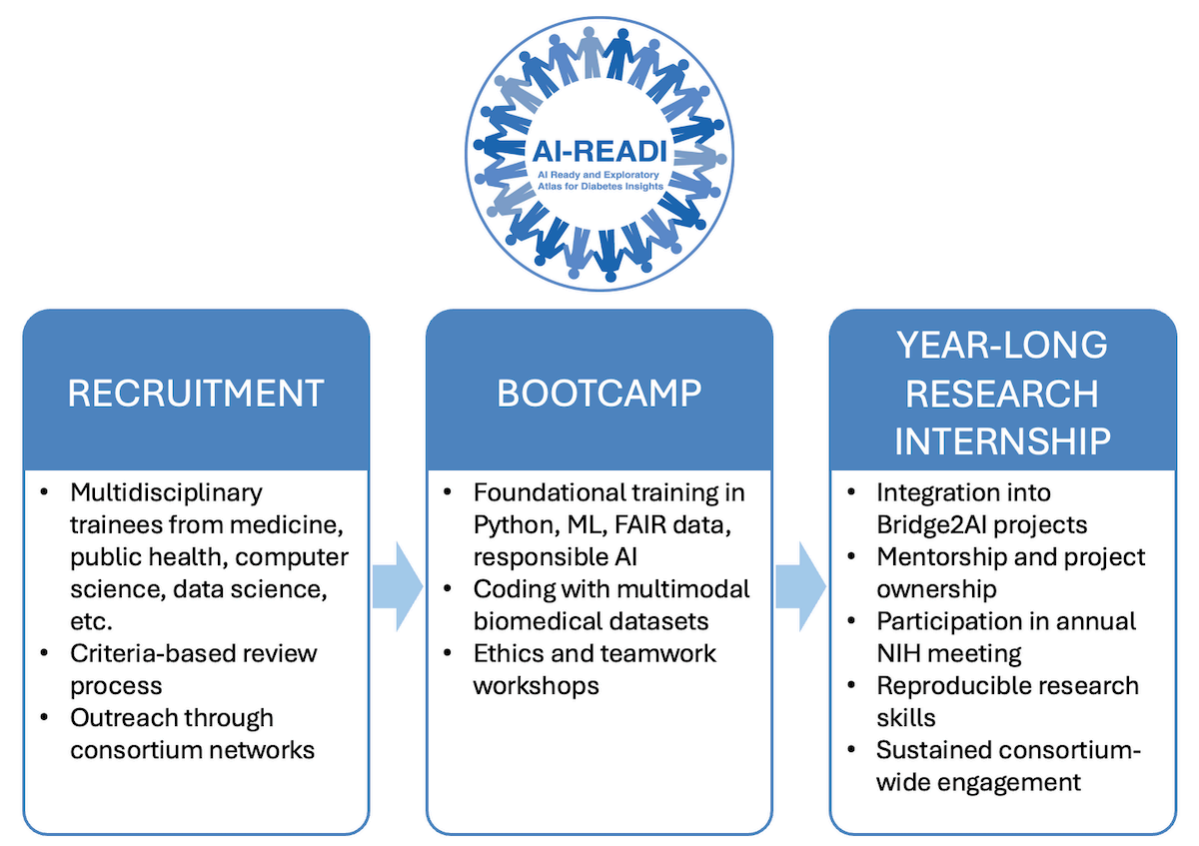
AI-READI skills and workforce development module within the NIH Bridge2AI initiative. AI: artificial intelligence; AI-READI: Artificial Intelligence–Ready and Exploratory Atlas for Diabetes Insights; Bridge2AI: Bridge to Artificial Intelligence; FAIR: findable, accessible, interoperable, and reusable; ML: machine learning; NIH: National Institutes of Health.

Beyond data generation, AI-READI integrates a 3-phase educational framework within the Bridge2AI ecosystem (Figure 1). This framework recruits multidisciplinary trainees, delivers an intensive 2-week AI/ML bootcamp, and transitions participants into a year-long mentored research internship. Together, these stages provide a continuum of learning that couples foundational instruction with sustained, project-based engagement, directly aligned with the consortium’s goal of building a diverse and AI-ready biomedical workforce.

Although a growing number of AI training programs exist—from massive open online courses to short-term institutional electives—many rely on generic or narrowly scoped datasets, offer limited interdisciplinary interaction, or lack sustained mentorship [10-12]. In contrast, the AI-READI Bootcamp was intentionally developed for trainees from diverse disciplines to collaborate on structured, domain-relevant biomedical datasets that reflect the complexity of real-world research. Led by faculty with experience in NIH- and National Science Foundation–funded training initiatives, the curriculum integrates seminars in ML, statistics, and responsible AI with notebook-based coding laboratories anchored in the AI-READI dataset.

Each cohort (Year 1=2023; Year 2=2024) was independently developed and iteratively refined in response to structured participant feedback. This feedback-driven design aligns with broader trends in AI education emphasizing modular content, scaffolded mentorship, and interdisciplinary collaboration [11-13].

This manuscript details the design and iteration of the AI-READI Bootcamp, summarizes participant characteristics and evaluation outcomes, and distills key lessons for institutions aiming to build inclusive, practice-oriented AI training programs in health care.

## Methods

### Ethical Considerations

This study involved the evaluation of an educational training program and analysis of aggregated, deidentified survey and program evaluation data collected as part of routine program assessment. In accordance with institutional and US federal guidelines (45 CFR §46) [14], formal institutional review board review was not required, as the project constituted educational program evaluation with minimal risk to participants and did not involve the collection of identifiable private information. Participation in surveys and program evaluations was voluntary. Participants were informed that their responses could be used for research and dissemination purposes and that participation or nonparticipation would not affect their standing in the program. Completion of the surveys was considered to imply informed consent. All data were analyzed in a deidentified and aggregated manner. No direct identifiers were collected or retained. Data were stored on secure, access-controlled institutional systems, and only study personnel had access to the data. Privacy and confidentiality were maintained throughout the study. Participants did not receive financial compensation for survey participation beyond the educational benefits associated with participation in the AI-READI Bootcamp.

### AI-READI Intern Recruitment and Bootcamp Participant Selection

Participants for the year-long AI-READI internship program were recruited from diverse academic and professional backgrounds, including computer science, engineering, medicine, public health, nursing, pharmacy, and allied health fields. Selection prioritized quantitative aptitude, scientific curiosity, and interdisciplinary interest rather than prior coding experience. Coursework in calculus, linear algebra, or statistics was preferred but not required for eligibility. Recruitment strategies included an informational brochure on the AI-READI website; dissemination through faculty web pages, journals, mailing lists, and social media; and outreach by alumni, mentors, and current trainees through word-of-mouth engagement.

Applicants completed an online application comprising educational history, research experience, a 750-word personal statement, and one faculty recommendation. Reviewers scored submissions on a 1‐5 scale across 4 domains: academic achievement, technical skills, research experience, and strength of recommendation. Top-ranked candidates received AI-READI–funded internship positions (6 in Year 1; 5 in Year 2), while additional high-scoring applicants were invited to participate in the bootcamp as unfunded trainees (11 in Year 1; 2 in Year 2).

### Bootcamp Structure and Educational Objectives

The AI-READI Bootcamp is a 2-week immersive, in-person educational program hosted annually at the University of California San Diego. It functioned as both the foundational training phase and the launchpad for the year-long mentored research internship. The curriculum emphasized collaborative, application-oriented learning tailored to participants’ diverse disciplinary backgrounds and varying levels of technical preparedness.

Educational objectives were to (1) develop proficiency in core programming workflows using Python, Jupyter Notebooks, and GitHub; (2) introduce foundational principles of AI/ML, including supervised and unsupervised learning; (3) provide hands-on experience through structured coding laboratories using multimodal, domain-relevant biomedical data; (4) promote reproducible and ethical research practices; and (5) foster interdisciplinary collaboration, critical thinking, and cohort cohesion.

The curriculum intentionally targeted multiple domains of Bloom’s taxonomy [15], integrating didactic instruction to build knowledge (cognitive), using applied coding to develop technical skills (psychomotor), and facilitating ethics discussions to promote responsible AI use (affective).

Participants completed approximately 80 hours of lectures, coding tutorials, and small-group mentorship sessions. Dormitory housing facilitated peer learning, collaborative debugging, and informal knowledge exchange. Instruction was led by a multidisciplinary faculty team spanning computer science, data science, medicine, public health, and ethics. Curriculum content was refined iteratively between cohorts in response to postbootcamp feedback (see Results).

### Participant Characteristics and Baseline Data Collection

Before the bootcamp, all participants completed a standardized intake form capturing demographic and educational information, including age, gender, highest degree attained, primary discipline, prior experience with programming languages (eg, Python, R, SQL), and self-reported exposure to AI/ML. These responses informed real-time instructional adjustments and enabled instructors to tailor laboratory groupings, pacing, and mentorship to each cohort’s skill profile.

### Postbootcamp Survey and Feedback Analysis

After completing the bootcamp, participants were invited to complete an anonymous evaluation survey assessing instructional quality and overall experience.

- Quantitative items: 7 core statements rated on a 5-point Likert scale (1=strongly disagree; 5=strongly agree) evaluated lecture usefulness, facility quality, instructor effectiveness, alignment with expectations, staff support, organizational quality, and overall enjoyment.
- Qualitative items: open-ended prompts solicited feedback on the most and least valuable components, suggested improvements, and logistical factors such as scheduling, pacing, and housing.

Quantitative data were summarized using descriptive statistics. Qualitative responses underwent independent dual-coder thematic analysis; discrepancies were resolved through discussion. Resulting insights informed iterative refinements to curriculum structure, pacing, and instructional methods between program years.

## Results

### Participant Characteristics

A total of 17 trainees participated in Year 1 and 7 in Year 2 of the AI-READI Bootcamp. As summarized in Table 1, participants came from varied academic and professional backgrounds, including ophthalmology, public health, pharmacology, neuroscience, engineering, and computer science. Educational attainment and programming experience also varied substantially, reflecting the program’s deliberate design to attract learners with strong quantitative potential regardless of prior coding experience.

**Table 1.** Participant characteristics of bootcamp cohorts.

Characteristic	Year 1 (n=17)	Year 2 (n=7)
Age (years), mean (SD)	33 (3.4)	32 (9.5)
Race, n (%)		
Asian	8 (47.1)	3 (42.9)
African American	3 (17.6)	2 (28.6)
White	4 (23.5)	1 (14.3)
Other	2 (11.8)	0 (0)
Sex, n (%)		
Male	9 (52.9)	2 (28.6)
Female	8 (47.1)	5 (71.4)
Highest degree, n (%)		
PhD or MD	15 (88.2)	4 (57.1)
MA, MS, or MPH	2 (11.8)	1 (14.3)
BA or BS	0 (0)	2 (28.6)
Funding, n (%)		
AI-READI–funded^a^	6 (35.3)	5 (71.4)
Non-AI-READI–funded	11 (64.7)	2 (28.6)
Disciplinary background, n (%)		
Ophthalmology	12 (70.6)	1 (14.3)
Public Health	1 (5.9)	1 (14.3)
Pharmacology	1 (5.9)	1 (14.3)
Neuroscience	1 (5.9)	0 (0)
Engineering	1 (5.9)	0 (0)
Biochemistry	1 (5.9)	0 (0)
Behavioral Science	0 (0)	1 (14.3)
Computer Engineering	0 (0)	1 (14.3)
Medicine	0 (0)	1 (14.3)
Molecular Biology	0 (0)	1 (14.3)
Familiarity with programming language, n (%)		
Python	3 (27.3)	4 (57.1)
R	5 (45.5)	4 (57.1)
SQL	1 (9.1)	2 (28.6)
JAVA	0 (0)	1 (14.3)
MATLAB	2 (18.2)	1 (14.3)
Julia	0 (0)	1 (14.3)
C	0 (0)	1 (14.3)
C++	3 (27.3)	1 (14.3)

a AI-READI: Artificial Intelligence–Ready and Exploratory Atlas for Diabetes Insights.

### Survey Feedback and Satisfaction Outcomes

Postbootcamp surveys were completed by 13 of 17 (76%) participants in Year 1 and 4 of 7 (57%) in Year 2. Across both cohorts, quantitative ratings were high, with notable improvements in Year 2 (Table 2). Year 2 participants assigned mean scores of 5.00 in 3 categories—instructor effectiveness, staff support, and overall enjoyment—while all other domains, including lecture usefulness, organizational quality, and facility adequacy, averaged between 4.50 and 4.75. No item received a mean rating below 4.0.

Qualitative feedback from Year 1 highlighted several key areas for improvement. Participants valued the conceptual depth of lectures but desired greater emphasis on applied content aligned with their upcoming research projects. Some noted that the larger cohort size made coding laboratories challenging to manage and recommended smaller groups to support individualized troubleshooting. Hands-on laboratories and mentorship—both from faculty and peers—were consistently described as the most valuable program components. Participants also emphasized the benefits of shared housing and peer interaction in fostering collaboration and community.

**Table 2.** Postbootcamp evaluation: comparison of Year 1 and Year 2.

Item	Year 1 (n=13)	Year 2 (n=4)
	Average (SD; range)	Average (SD; range)
The lectures were helpful to my learning and development	4.46 (1.05; 2.00-5.00)	4.75 (0.50; 4.00-5.00)
The bootcamp facility was in an accessible location and adequate	4.46 (1.00; 2.00-5.00)	4.75 (0.50; 4.00-5.00)
The instructors helped me understand the subject matter	4.31 (1.11; 1.00-5.00)	5.00 (0.00; 5.00-5.00)
The bootcamp met my educational needs and expectations	4.31 (1.07; 1.00-5.00)	4.75 (0.50; 4.00-5.00)
I had adequate support from the program staff and faculty	4.46 (1.20; 1.00-5.00)	5.00 (0.00; 5.00-5.00)
The bootcamp was well organized	4.23 (1.24; 1.00-5.00)	4.50 (0.58; 4.00-5.00)
I enjoyed the bootcamp overall	4.46 (1.00; 2.00-5.00)	5.00 (0.00; 5.00-5.00)

The redesigned Year 2 curriculum addressed many of these concerns. Participants emphasized the benefits of earlier integration of the AI-READI dataset, closer alignment between instructional content and research projects, and strengthened peer collaboration. Several respondents noted that receiving materials and agendas in advance would have enhanced preparation for more technical sessions. Overall, Year 2 participants described the bootcamp as a strong foundation for subsequent research activities, increasing both confidence and competence.

Together, these findings validate the bootcamp’s iterative design and demonstrate that refinements in Year 2 enhanced the learning experience while preserving core strengths in applied instruction, mentorship, and interdisciplinary collaboration.

### Curriculum Iteration and Structure

The curriculum was iteratively refined between Year 1 and Year 2 based on participant feedback and faculty debriefings. Year 1 focused on establishing foundations in Python workflows, core ML algorithms, and introductory discussions on ethics and bias. In Year 2, the instructional team implemented several structural and pedagogical updates to enhance applied learning and mentorship. The cohort size was reduced to achieve an approximately 1:2 faculty-to-student ratio, and modules on Git/GitHub version control and environment setup were moved earlier to reinforce reproducibility. Structured, domain-relevant data from the AI-READI project—including retinal images and clinical variables—were integrated throughout the curriculum, allowing trainees to engage directly with multimodal biomedical data that mirrored the complexity of biomedical research workflows.

Additional modules were introduced in Year 2 to align with evolving learner needs and faculty expertise. Mini-seminars on FAIR data principles, AI-READI schema design, and agile project management helped participants navigate the practical aspects of large-scale dataset curation. A dedicated half-day session on large language models introduced transformer architectures and encouraged discussion of the opportunities and limitations of generative AI in health care. The Year 2 capstone project centered on fine-tuning RETFound, a retina-specific foundation model for institutional classification of retinal images, fostering critical reflection on domain generalizability and algorithmic bias.

Tables3^5^ summarize the curriculum’s progression from foundational programming to applied AI/ML methods, highlighting key updates, instructional content, and the shift toward hands-on, clinically relevant learning experiences.

**Table 3.** Summary of curriculum refinements between Year 1 and Year 2 of the AI-READI^a^ Bootcamp.

Dimension	Year 1 focus	Year 2 iteration and rationale
Programming foundations	Introduction to Python and Jupyter via guided exercises	Added pandas and real-world data operations to support independent analysis
Tools and environment	Introduced GitHub and IDEs^b^ for version control	Moved earlier to establish reproducibility from the start
Machine learning concepts	Covered regression, SoftMax, convolutional neural networks, and backpropagation	Added large language models and expanded gradient descent laboratories
Data science techniques	Applied principal component analysis, K-means, and spectral clustering to face images	Expanded to exploratory data analysis, digital signal processing, and feature extraction
Applied learning	Face clustering and glucose modeling laboratories	Shifted to retinal image analysis using the AI-READI dataset
Ethics and fairness	Discussed racial bias in pain expression	Broadened to data pitfalls and fairness across AI^c^ pipelines
Clinical integration	Minimal use of clinical data	Incorporated AI-READI clinical variables and retinal images
Student engagement	Lunch talks and informal discussions	Added structured mini-seminars and interactive sessions for peer learning

a AI-READI: Artificial Intelligence–Ready and Exploratory Atlas for Diabetes Insights.

b IDE: integrated development environment.

c AI: artificial intelligence.

**Table 4.** Year 1 AI-READI^a^ Bootcamp curriculum.

Day	Session topics	Format
1	Bootcamp orientation; introduction to Python, Jupyter notebooks	Lecture + hands-on laboratories
2	Python, IDEs^b^, Jupyter workflows	Lecture + coding practice
3	Introduction to machine learning, perceptrons, gradient descent; logistic and SoftMax regression	Lecture + laboratories
4	Backpropagation, deep learning fundamentals; representation learning	Lecture + laboratories + discussion
5	Convolutional neural networks	Lecture + laboratories
6	GitHub version control; introduction to pandas	Lecture + coding
7	Linear algebra review; regression models; regression laboratories	Lecture + regression laboratories
8	Principal component analysis, face laboratories, K-means, spectral clustering	Lecture + laboratories
9	Clustering laboratories; discussion on racial bias in data; pitfalls in data science	Lecture + laboratories + ethics discussion
10	Digital signal processing; glucose laboratories; closing reflections	Lecture + laboratories + closing

a AI-READI: Artificial Intelligence–Ready and Exploratory Atlas for Diabetes Insights.

b IDE: integrated development environment.

**Table 5.** Year 2 AI-READI^a^ Bootcamp curriculum.

Day	Session topics	Format
1	Introduction to Python, Jupyter, IDEs^b^; GitHub version control	Lecture + hands-on laboratories
2	Python pandas, joining datasets, exploratory data analysis	Lecture + coding exercises
3	Correlations, health sheet overview, data visualization	Lecture + laboratories
4	Digital signal processing, feature extraction, basic image processing	Lecture + laboratories
5	Linear algebra, regression models (linear, nonlinear, ridge, lasso)	Lecture + regression laboratories
6	Principal component analysis (PCA), image alignment, introduction to clustering	Lecture + PCA laboratories
7	Clustering (K-means), pitfalls in data science	Lecture + laboratories + discussion
8	Machine learning introduction, perceptrons, logistic/SoftMax regression	Lecture + gradient descent laboratories
9	Backpropagation, deep learning, representation learning, machine learning best practices	Lecture + eigenface laboratories
10	Convolutional neural networks, introduction to large language models	Lecture + coding demos

a AI-READI: Artificial Intelligence–Ready and Exploratory Atlas for Diabetes Insights.

b IDE: integrated development environment.

## Discussion

### Principal Results and Learner Outcomes

Quantitative and qualitative outcomes demonstrate that the AI-READI Bootcamp effectively delivered foundational AI/ML education to multidisciplinary biomedical trainees, yielding consistently high satisfaction and confidence across 2 consecutive years. In Year 2, mean postbootcamp ratings improved across all domains—from 4.23‐4.46 (Year 1: n=13) to 4.50-5.00 (Year 2: n=4)—with 3 categories (instructor effectiveness, staff support, and overall enjoyment) achieving perfect 5.00 scores. These findings align with Kirkpatrick’s training evaluation model (Levels 1 and 2 outcomes), reflecting strong learner satisfaction and perceived knowledge gains, while qualitative feedback suggested enhanced confidence and readiness for independent research (Level 3 outcome).

### Bridging Disciplinary Divides in AI/ML Education

As AI continues to transform biomedical research and clinical practice, a persistent skills gap remains between data scientists and clinicians [16-18]. Engineers may lack clinical context, whereas physicians often have limited exposure to algorithmic reasoning and data analytics—constraints that can hinder translational innovation and collaboration.

The AI-READI Bootcamp was intentionally designed to bridge this divide by uniting trainees from medicine, neuroscience, computer science, public health, and pharmacology under a shared mentorship model spanning technical and clinical faculty. This approach reflects best practices identified in recent AI curriculum review [5,11,12] and parallels pedagogical strategies in health professions education—such as interprofessional and team-based learning—that foster cross-disciplinary problem-solving and shared understanding [19].

### Building Engagement Through Iterative Refinement

Iterative curriculum refinement was central to sustaining engagement and relevance. Key adjustments between Year 1 and Year 2—including smaller cohort size, earlier integration of AI-READI datasets, and increased time for small-group coding—were guided directly by participant feedback. Year 2 trainees highlighted the benefits of multimodal biomedical data, individualized mentorship, and peer collaboration as core strengths.

Anchoring abstract AI/ML concepts in domain-specific datasets proved particularly effective. By analyzing fundus photographs and structured clinical variables from the AI-READI dataset, participants investigated issues such as site-level variability and domain shift, deepening their understanding of real-world data challenges. This experiential approach aligns with educational frameworks emphasizing authentic data environments and iterative feedback [20], supporting evidence that short-format programs can achieve substantial impact when paired with applied learning and sustained mentorship.

### Situating the Bootcamp in the National AI Training Ecosystem

The AI-READI Bootcamp contributes to a growing ecosystem of NIH-supported initiatives advancing the biomedical AI/ML workforce. Within the Bridge2AI program, AI-READI complements other DGPs—including VOICE, which hosts AI summer schools and hackathons on precision public health, and CHoRUS, which provides continuing medical education–accredited clinical AI workshops on dataset curation, pair programming, and mentored laboratories [9].

Beyond Bridge2AI, the AIM-AHEAD Consortium extends these efforts through part-time fellowships, faculty development programs, and mentored research opportunities aimed at graduate students, health care professionals, and community partners [21]. Parallel innovations are emerging at the institutional level: Stanford University engages students in interdisciplinary teams applying ML to clinical challenges; the Duke Institute for Health Innovation pairs medical trainees with data scientists; and programs at the University of Florida and Carle Illinois College of Medicine integrate clinician-engineer coteaching models. Collectively, these efforts underscore the increasing national and institutional commitment to embedding AI within health professions education, though many remain short-term or elective in scope.

### Distinctive Features and Broader Applicability

Although numerous national initiatives—such as AIM-AHEAD, VOICE, and CHoRUS—have expanded AI/ML education through workshops and fellowships, the AI-READI Bootcamp occupies a distinctive niche within this ecosystem. By embedding a 2-week immersive experience within a year-long mentored research internship, it integrates foundational instruction with sustained, project-based engagement. In addition to mastering core AI/ML methods and completing mentored research projects, participants contribute to consortium-wide Bridge2AI initiatives focused on data standardization, FAIR and ethical data practices, biorepository optimization, and workforce development. This multifaceted structure positions the AI-READI Bootcamp as both a pilot and a scalable framework for cultivating interdisciplinary expertise in biomedical AI.

### Lessons Learned and Recommendations

Our experience designing and refining the AI-READI Bootcamp suggests several important lessons for future initiatives. Modular, scaffolded content enables learners with varying backgrounds to progress in parallel. The use of curated, domain-relevant datasets grounds abstract concepts in applied contexts, fostering deeper engagement. Participants valued the theoretical framing but reported that they learned most effectively through practical, hands-on components, suggesting future bootcamps should emphasize applied coding while keeping lectures concise and focused.

Equally important are the program’s structural features. Maintaining a low faculty-to-student ratio supports real-time troubleshooting and individualized feedback. Embedding bootcamps into longitudinal research structures promotes meaningful skill transfer and project ownership. Structured and informal peer support—through shared housing, collaborative debugging, and group presentations—strengthens technical skills, enhances problem-solving, and builds lasting professional networks. These practices align with curriculum frameworks emphasizing structure, assessment, real-world alignment, and longitudinal mentorship [5,10-12].

### Scalability, Replication, and Sustainability

The AI-READI Bootcamp model was intentionally designed for scalability and replication through a modular curriculum organized into discrete instructional units. In alignment with FAIR and open science principles, all lectures, laboratories, and onboarding materials are publicly available on GitHub, enabling other institutions to adapt content to their own technical and educational contexts. The program’s structure, which pairs short-term immersive instruction with a longitudinal mentored research experience, offers a reproducible framework that can be integrated into diverse training pipelines, including graduate education, residency programs, and interdisciplinary research initiatives. The AI-READI model also promotes long-term sustainability through its open educational resources within the Bridge2AI consortium, embedded mentorship networks, and continued dissemination of curricular updates and trainee outcomes across the Bridge2AI community.

### Limitations and Future Directions

This study is limited by the absence of standardized, performance-based assessments, which are necessary to measure knowledge retention and applied competency. Postbootcamp evaluations for both years relied on self-reported survey data, which are subject to response and recall bias and may not directly reflect objective skill acquisition. In addition, the study is limited by its single-site implementation and small sample size (Year 1: n=17; Year 2: n=7), which may affect generalizability. The Year 2 postbootcamp ratings, while high, are based on only 4 respondents.

To address these gaps, future iterations will incorporate pre- and postbootcamp knowledge assessments and coding exercises aligned with Kirkpatrick Level 2 outcomes to objectively measure learning gains. Longitudinal tracking of trainee outputs such as publications, presentations, and continued engagement in AI-related research will further assess sustained impact. Broader dissemination of the curriculum and collaboration across Bridge2AI partner DGPs may also enhance reproducibility and external validation of outcomes. We also plan to strengthen prebootcamp onboarding (Table 6) through structured preassessment materials, curated readings, and practice exercises to improve baseline preparedness and maximize in-person learning.

Through these refinements, the AI-READI Bootcamp aims to evolve from a formative, single-site pilot to a scalable, high-impact model for interdisciplinary AI/ML education in biomedicine. To promote transparency and adoption, all bootcamp materials, readings, and onboarding instructions are openly available via the AI-READI Bootcamp GitHub repository (Table 6).

**Table 6.** Prebootcamp onboarding module and recommended readings.

Category and resource	Details and access
Core online text	
Dive into Deep Learning (D2L)	Website [22]
Bootcamp GitHub repository	
AI-READI^a^ Bootcamp GitHub	Web page on GitHub [23]
Readings (author, year)	
Berisha et al, 2021 [24]	Available on Bootcamp GitHub Readings page [23]
Bishop, 2006 [25]	Chapter 1; introduction to chapter 9 & section 9.1; introduction to chapter 12 & section 12.1; Appendix C
Ezer & Whitaker, 2019 [26]	Available on Bootcamp GitHub Readings page [23]
Obermeyer et al, 2019 [27]	Available on Bootcamp GitHub Readings page [23]
Rumelhart et al, 1987 [28]	Available on Bootcamp GitHub Readings page [23]
Strang, 2016 [29]	Also see YouTube lectures [30]
Wilkinson et al, 2016 [31]	Available on Bootcamp GitHub Readings page [23]
Zou & Schiebinger, 2018 [32]	Available on Bootcamp GitHub Readings page [23]
Helpful links	
Introduction to Python	Available on Bootcamp GitHub Readings page [23]
Git terminology	Available on Bootcamp GitHub Readings page [23]
Setting up Git	Available on Bootcamp GitHub Readings page [23]

a AI-READI: Artificial Intelligence–Ready and Exploratory Atlas for Diabetes Insights.

## Abbreviations

AI: artificial intelligence
AI-READI: Artificial Intelligence–Ready and Exploratory Atlas for Diabetes Insights
Bridge2AI: Bridge to Artificial Intelligence
DGP: data generation project
FAIR: findable, accessible, interoperable, and reusable
ML: machine learning
NIH: National Institutes of Health
